# Superior vena cava syndrome associated with sarcoidosis: a very rare case of veinous parietal involvement

**DOI:** 10.1093/omcr/omaf016

**Published:** 2025-04-28

**Authors:** Khaoula Boumeriem, Kaoutar Imrani, Iliass Bourekba, Nabil Moatassim Bilah, Itimad Nassar

**Affiliations:** Central radiology department, Ibn sina university hospital of Rabat, Rabat 90 000, Morocco; Central radiology department, Ibn sina university hospital of Rabat, Rabat 90 000, Morocco; Central radiology department, Ibn sina university hospital of Rabat, Rabat 90 000, Morocco; Central radiology department, Ibn sina university hospital of Rabat, Rabat 90 000, Morocco; Central radiology department, Ibn sina university hospital of Rabat, Rabat 90 000, Morocco

**Keywords:** superior vena cava syndrome, sarcoidosis, granulomatosis

## Abstract

Sarcoidosis, a systemic disorder characterized by non-caseating granulomas, poses diagnostic challenges due to its diverse manifestations. We present a rare case of superior vena cava syndrome (SVCS) secondary to most likely granulomatous involvement in a patient with mediastinal sarcoidosis. While SVCS typically arises from extrinsic compression or thrombosis, our case highlights the importance of considering inflammatory thickening of the SVC wall as a potential cause, requiring appropriate treatment.

## Introduction

Sarcoidosis is a multisystemic non-caseating granulomatous disorder of unknown etiology, in which pulmonary involvement is the most common and often revealing manifestation of the disease. However, extra-pulmonary manifestations are diverse and sometimes isolated, venous vascular involvement, in particular, is rare, typically attributed to venous compression or thrombosis due to hypercoagulability [1–3]. Nevertheless, it is essential not to overlook granulomatous inflammatory venous lesions, as management treatment differs [4, 5].

Here, we present the case of a 54-year-old female patient in whom probable granulomatous infiltration of the superior vena cava (SVC) was discovered during the investigation of a newly acquired superior vena cava syndrome (SVCS). Through this case report, we aim to shed light on this rare form of inflammatory vascular involvement in sarcoidosis and its implications for diagnosis and management.

## Case report

A 54-year-old female patient, with a history of mediastinal sarcoidosis (Diagnosed based on a set of clinical and biological findings, and not treated due to the initial absence of pulmonary or extrapulmonary lesions), presented with cervicofacial edema accompanied by distended cervical veins for the past two weeks. She was admitted for exploration of a superior vena cava syndrome.

**Figure 1:**
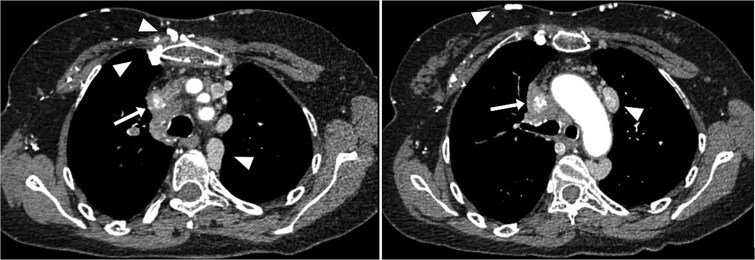
Axial CT scan with contrast injection showing parietal thickening of the SVC leading to luminal narrowing (arrow). Collateral venous circulation is also noted, indicating obstructed venous drainage and thus superior vena cava syndrome (arrowheads).

**Figure 2:**
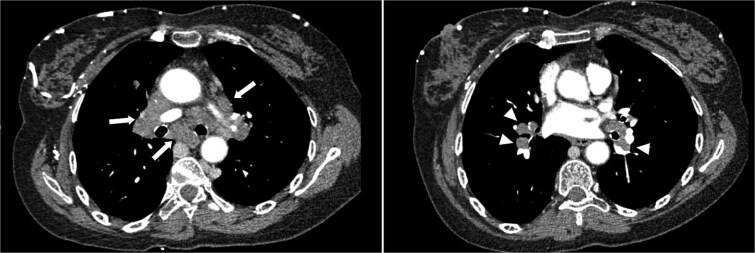
Axial CT scan showing multiple mediastinal (arrows) and hilar (arrowheads) lymphadenopathies, with no venous compression.

**Figure 3:**
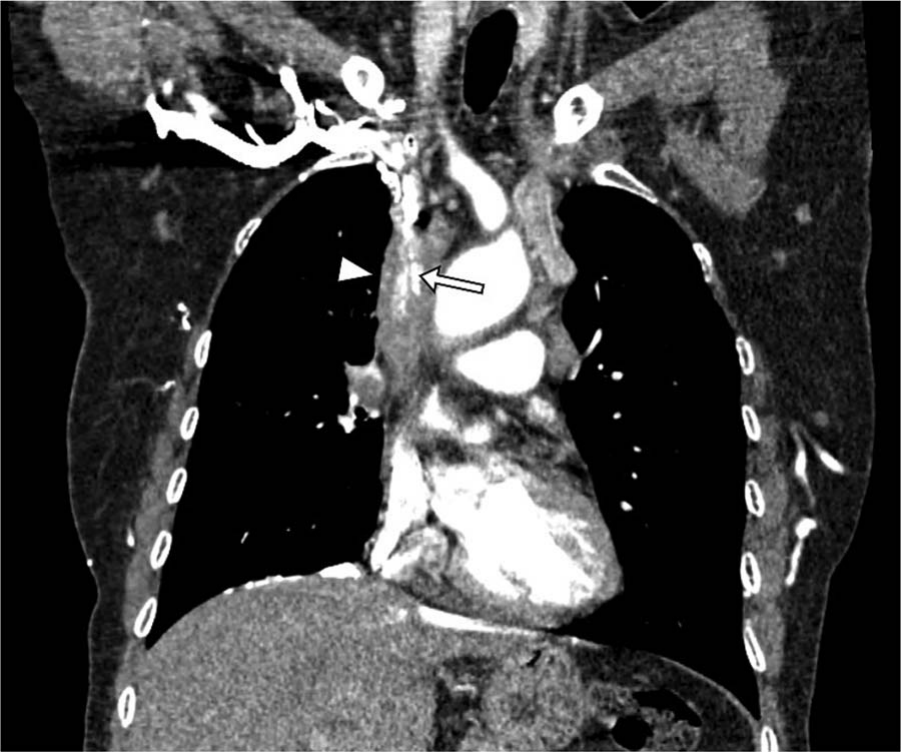
Coronal slices providing a clearer visualization of the parietal thickening (arrowheads) and the progressive reduction in vascular lumen until complete occlusion (arrow).

**Figure 4:**
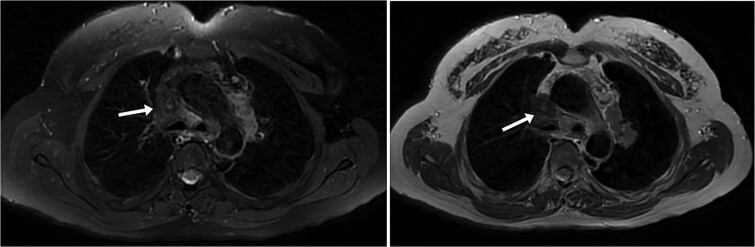
MRI T2-weighted and T2 FS sequences allowing for a more detailed analysis of the parietal thickening of the SVC. It is noted that there is no endoluminal thrombus or compressive lymphadenopathy adjacent to the SVC.

**Figure 5:**
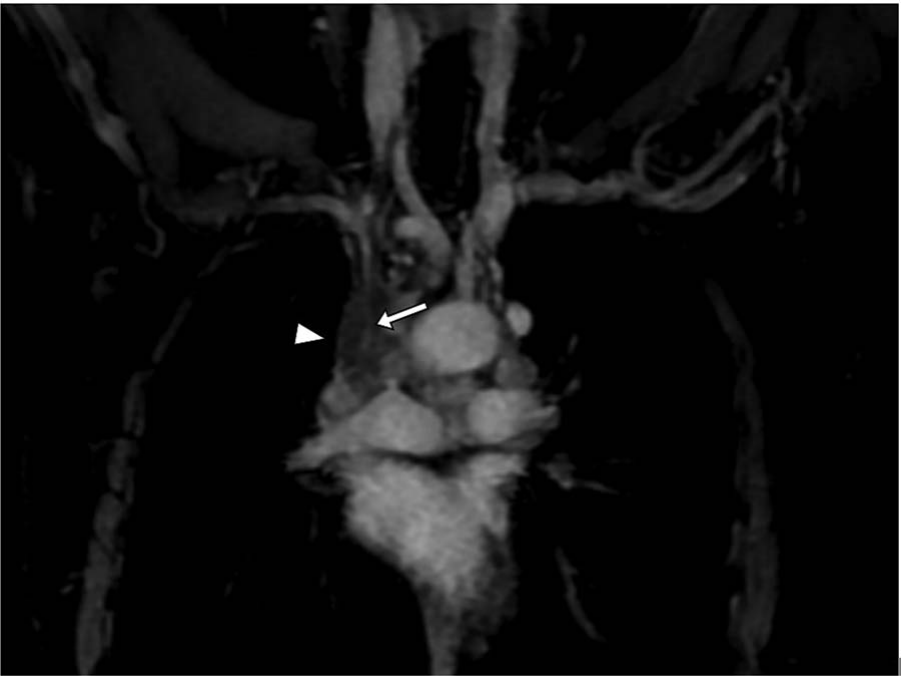
Coronal T1 FS GD + sequence providing excellent visualization of the parietal infiltration of the SVC (arrowheads) and its impact on the venous lumen (arrow).

A contrast-enhanced thoracic computed tomography (CT) scan revealed complete occlusion of the superior vena cava with notable collateral venous circulation. Multiple mediastinal lymphadenopathies were also observed, although non-compressive (Fig. 2). In addition to the SVCS, a thickening of venous segments proximal to the thrombosis was observed (Fig. 1 and Fig. 3). Consequently, additional magnetic resonance imaging (MRI) assessment was requested to provide a more detailed analysis of the superior vena cava wall structure. The MRI findings demonstrated wall thickening of the superior vena cava (Fig. 4), accompanied by progressive reduction in vascular lumen, resulting in complete occlusion, resembling a ‘rat’s tail sign’ appearance (Fig. 5).

Laboratory investigations revealed an inflammatory syndrome characterized by elevated C-reactive protein (CRP) and erythrocyte sedimentation rate (ESR). The subsequent diagnostic workup excluded tuberculosis, as both the Quantiferon-TB® and GeneXpert MTB tests were negative. Additionally, lymphoma and pulmonary malignancy were ruled out, with no hyperlymphocytosis observed in the blood count and the CT scan showing no pulmonary lesion. Based on these findings, probable granulomatous involvement of the superior vena cava was considered the most likely diagnosis, leading to the initiation of treatment without a biopsy due to the high risk of hemorrhage associated with the procedure.

The patient received anticoagulant therapy, initially with low molecular weight heparin at a therapeutic dose, followed by a switch to oral vitamin K antagonists for 6 months. The patient was also placed on prednisolone 1 mg/kg for 4 weeks, with follow-up of the ESR and CRP, which nearly normalized after 3 weeks. A follow-up CT scan six months later showed re-permeabilization of the superior vena cava.

## Discussion

Sarcoidosis is a multi-systemic pathology of unknown origin, characterized by non-caseating granulomatous inflammation affecting multiple organs. It typically affects young adults, with a particular predilection for the lungs, joints, and reticuloendothelial system. The disease is often revealed by respiratory manifestations, such as chronic cough and dyspnea. The diagnosis of sarcoidosis is based on a combination of clinical and radiological findings, corroborated by histological findings in cases of atypical presentation [1].

Vascular involvement in sarcoidosis is rare but well-known, usually manifesting as vasculitis affecting both small vessels, causing typical skin and retinal lesions, and large vessels, with a preference for pulmonary arteries, aorta, and supra-aortic trunks [2]. Moreover, numerous cases of Takayasu syndrome—sarcoidosis association have been described [3].

However, venous involvement is extremely rare. Most reported cases of venous occlusion in the literature were due to extrinsic compression by lymphadenopathy or thrombophlebitis, given the demonstrated link between sarcoidosis and hypercoagulability. The number of cases in which venous wall thickening is described on imaging remains very limited; only three cases are reported in the literature to our knowledge, all involving the superior vena cava (SVC) and innominate vein [4, 5]. This might be attributed to the fact that diagnosis is generally made by CT scan, which often cannot differentiate between venous wall thickening and endoluminal thrombosis, especially since histological sampling is usually unfeasible.

Imaging remains the only means to detect venous wall involvement. While ultrasound may visualize wall thickening and assess vein patency, its utility is limited in cases where the SVC is affected, as seen in all reported cases. Therefore, cross-sectional imaging is essential. Although CT scan theoretically can visualize venous wall thickening, MRI with its excellent contrast resolution is the most suitable examination. It typically reveals a non-specific inflammatory venous wall thickening, gradually reducing vein lumen with or without total occlusion. The wall appears hypointense on T2-weighted imaging and may demonstrate late enhancement [5, 6]. The appearance closely resembles that seen in superior vena cava involvement in Behçet’s disease [7].

The prognosis is generally related to the severity of pulmonary and arterial involvement in sarcoidosis. Venous involvement rarely leads to serious complications and seems to usually responds well to medical treatment. However, the data remain limited, making it difficult to have a clear understanding of the overall prognosis for this type of involvement [5, 6].

The differential diagnosis includes all systemic diseases capable of causing venous involvement. As sarcoidosis rarely presents with this type of lesion, the diagnosis is generally considered when other more typical signs are present, notably mediastinal lymphadenopathy and interstitial pulmonary lesions [2, 3].

Given the rarity of cases, there’s no consensus regarding the therapeutic management. However, effective anticoagulation should always be combined with corticosteroids or immunosuppressants since the mechanism of the SVCS is secondary to inflammatory involvement of the vein.
